# A Novel Application of Injectable PRF for Superior Sulcus Hollowing

**DOI:** 10.1111/jocd.70666

**Published:** 2026-01-20

**Authors:** Nese Arslan, Alperen Bahar, Ahmet Alp Bilgic, Şule Barman Kakil

**Affiliations:** ^1^ Department of Ophthalmology Saglik Bilimleri Universitesi Diskapi Yildirim Beyazit Egitim ve Arastirma Hastanesi Ankara Turkey; ^2^ Department of Ophthalmology SBU Ulucanlar Goz Egitim Ve Arastirma Hastanesi Ankara Turkey; ^3^ Department of Ophthalmology FEBO, Mustafa Kemal Universitesi Tayfur Ata Sokmen Tip Fakultesi Antakya Hatay Turkey

**Keywords:** autologous biologic filler, deep superior sulcus, injectable platelet‐rich fibrin (i‐PRF), periocular rejuvenation

## Abstract

**Background:**

Superior sulcus hollowing is a frequent esthetic concern, commonly managed with synthetic fillers or fat grafts; however, these approaches carry potential risks and costs. Injectable platelet‐rich fibrin (i‐PRF), an autologous regenerative biomaterial, has shown promise in dermatologic and dental applications, but its use for periocular volume restoration remains unexplored.

**Aims:**

To evaluate the efficacy, safety, and patient satisfaction of i‐PRF injections for the correction of superior sulcus hollowing.

**Patients/Methods:**

This single‐center retrospective study included 24 eyes of 12 patients treated with i‐PRF between 2021 and 2024. Sulcus depth was measured at baseline and at 3 and 6 months posttreatment. Subjective satisfaction was assessed using a binary scale. i‐PRF was prepared using a low‐speed centrifugation protocol and injected suborbicularis in three sessions spaced 1 week apart.

**Results:**

A significant reduction in superior sulcus depth was observed from baseline to 3 months (13.3 ± 2.8 mm to 9.2 ± 3.7 mm; *p* = 0.002), followed by mild regression at 6 months (10.5 ± 3.3 mm). Patient satisfaction was high, with 18 out of 24 eyes (75%) reporting favorable outcomes at 3 months. No adverse effects were recorded.

**Conclusions:**

This first clinical report of i‐PRF for upper eyelid hollowing suggests that it may serve as a safe, autologous, and minimally invasive alternative for superior sulcus volume restoration. Further prospective studies with standardized outcome measures are warranted to validate these findings.

## 1 Introduction

**FIGURE 1:**
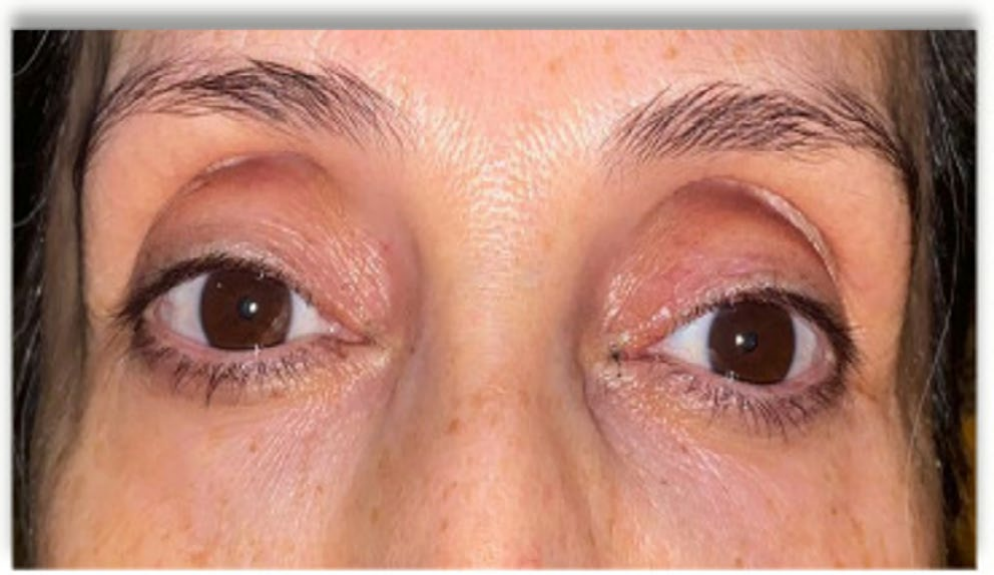
Pretreatment appearance of a patient with superior sulcus hollowing. The pronounced concavity of the upper eyelid sulcus, particularly in the medial and central compartments, contributes to a fatigued, aged appearance.

Superior sulcus hollowing, commonly referred to as a “sunken upper eyelid,” is primarily the result of age‐related volume loss in the periorbital region. Additional contributing factors include dehydration, sleep deprivation, trauma, or excessive fat removal during upper eyelid surgery (blepharoplasty) [1]. Anatomically, loss of orbital fat in the medial and central compartments, together with volume depletion at the orbital rim, creates a visible concavity in the superior sulcus (SS) (Figure 1). This change produces a fatigued or aged appearance, prompting many individuals to seek esthetic correction.

Several interventions have been employed to address superior sulcus hollowing, including hyaluronic acid (HA) fillers, autologous fat grafting, and dermis‐fat implantation [2, 3]. However, each method has limitations, such as high cost, procedural invasiveness, and potential complications including infection or, rarely, blindness.

Platelet‐rich fibrin (PRF), a second‐generation autologous platelet concentrate first introduced by Choukroun et al. in 2001 [4], consists of a dense fibrin matrix enriched with platelets, leukocytes, and growth factors [5]. Unlike platelet‐rich plasma (PRP), PRF is produced by single‐step low‐speed centrifugation without anticoagulants, allowing natural platelet activation and the gradual release of regenerative cytokines. In 2014, an injectable form—injectable PRF (i‐PRF)—was developed by adjusting centrifugation parameters and using plastic tubes, which extend its liquid working time to approximately 15–20 min [6]. This enables in situ fibrin polymerization after injection, enhancing local regenerative effects. Compared with PRP, i‐PRF demonstrates superior capacity for cell migration, proliferation, and angiogenesis due to its natural activation process and absence of additives [7, 8].

While PRP has been used in ophthalmology for ocular surface disorders, the application of PRF in this field remains limited. A few studies have explored its role in pterygium surgery [9, 10], but to our knowledge, no clinical study has evaluated i‐PRF for the treatment of superior sulcus hollowing. The aim of the present study was to assess the efficacy, safety, and patient satisfaction of i‐PRF injections for this indication.

## 2 Material and Methods

This single‐center retrospective cohort study with prospective follow‐up included 24 eyes of 12 consecutive patients treated for superior sulcus hollowing between January 2021 and December 2023. Ethical approval was obtained in June 2023 (No: 2023–292), covering both retrospective data review and prospective follow‐up through June 2024. All procedures were performed in a university oculoplastic clinic by the same surgeon. Written informed consent for both treatment and clinical photography was obtained from all participants.

**FIGURE 2:**
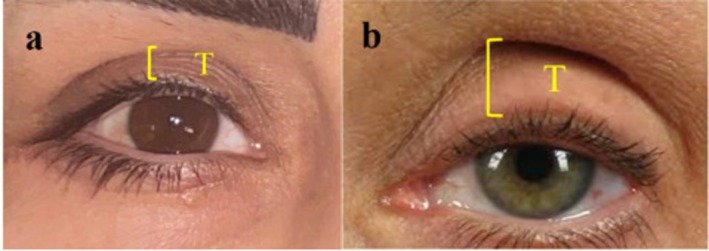
Measurement of pretarsal skin show (T) in two patients. (a) Example of a wide pretarsal area (> 7 mm) before treatment. (b) Another case with prominent sulcus depth. The vertical yellow bracket represents the measured distance between the upper eyelid margin and the superior orbital rim in primary gaze.

The depth of the superior sulcus was assessed by measuring the vertical distance between the superior orbital rim and the upper eyelid margin in primary gaze (Figure 2). Inclusion criteria were: (1) pretarsal skin show > 7 mm [11], (2) no prior upper eyelid filler or fat grafting, and (3) willingness to undergo i‐PRF treatment. Exclusion criteria included: (1) systemic or ocular disease, (2) prior orbital or eyelid surgery, (3) use of topical ophthalmic medications, and (4) incomplete follow‐up data. The primary endpoint was the change in superior sulcus depth from baseline to 3 months, with secondary endpoints including 6‐month depth and patient‐reported satisfaction.

For i‐PRF preparation, 10 mL of venous blood was collected from the antecubital vein into sterile plastic tubes without anticoagulant (Vacutainer, Becton Dickinson, USA). Samples were centrifuged immediately at 700 rpm for 3 min (~60 × g RCF) using a DLAB 0506 tabletop centrifuge. The upper yellow‐orange plasma layer was aspirated using an 18G blunt needle with a Luer‐lock syringe and injected within 15 min of preparation to prevent early fibrin polymerization. This protocol was based on the low‐speed i‐PRF method described by Mourão et al. [12] The site of deepest sulcus was marked with the patient in an upright position and in primary gaze. Topical anesthesia was applied using a combination of 2.5% prilocaine and 2.5% lidocaine creams. i‐PRF was injected into the suborbicularis oculi plane, targeting the area of maximum concavity in the superior sulcus. Each eye received 0.75–1.0 mL, divided into two suborbicularis boluses using a 30G needle (0.3 × 13 mm). Topical anesthesia was applied (2.5% prilocaine + 2.5% lidocaine) 30 min prior to injection. Injections were performed with the patient in an upright position to optimize visualization of the sulcus and minimize vascular risk. Care was taken to avoid the infraorbital artery and supraorbital neurovascular structures.

**FIGURE 3:**
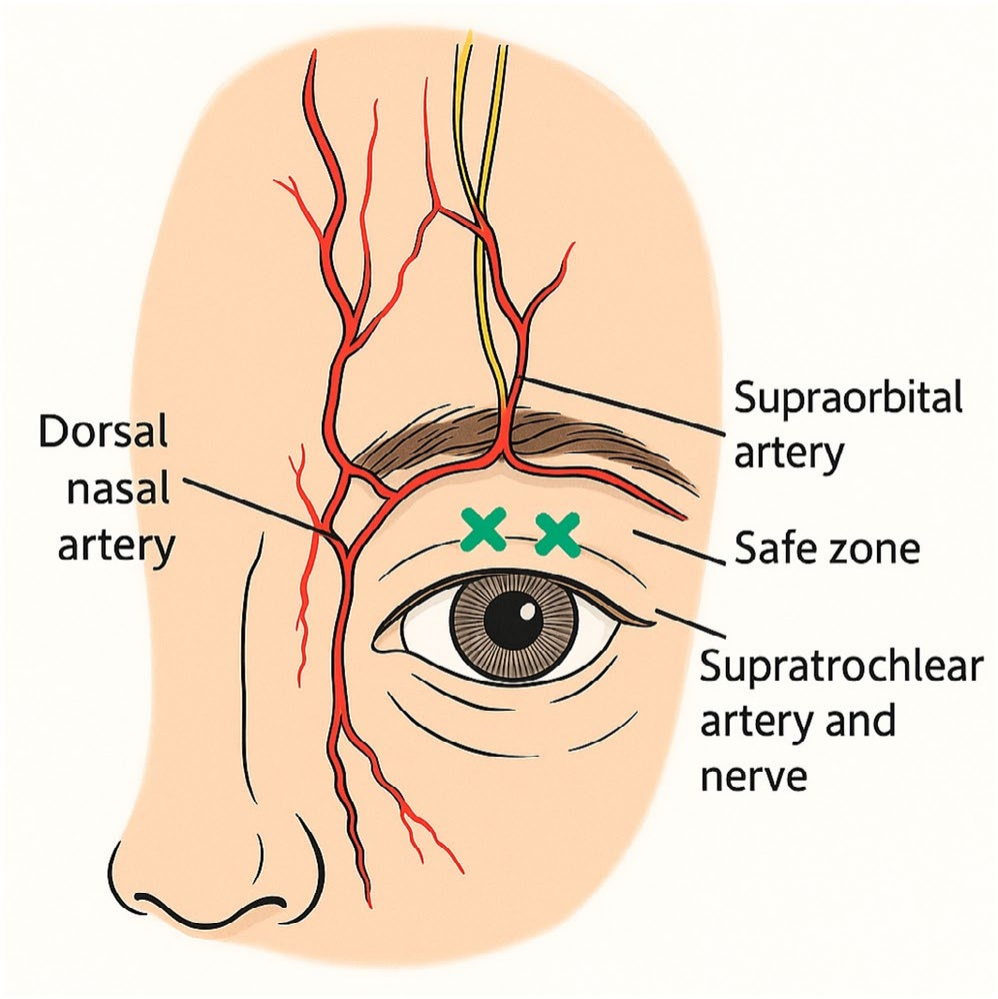
Illustration of the suborbicularis i‐PRF injection technique for upper eyelid rejuvenation. The green “X” marks indicate the recommended safe injection points along the upper eyelid lid crease, avoiding the supraorbital and supratrochlear arteries. The injection is performed with a 30G needle, with approximately 0.15–0.2 mL per site, totaling 1.5–2 mL for both eyes.

A new procedural diagram (Figure 3) illustrates the typical injection points. Minor adverse events included transient edema and mild bruising in 2 patients, resolving spontaneously within 3 days. No serious complications were observed.

## 3 Statistical Analysis

Statistical analysis was performed using IBM SPSS Statistics version 26.0 (IBM Corp., Armonk, NY, USA). Changes in superior sulcus depth at 3 and 6 months compared to baseline were evaluated using two‐tailed paired *t*‐tests, with *p* < 0.05 considered statistically significant. Data are expressed as mean ± standard deviation (SD). Intrapatient variability was assessed by comparing bilateral measurements in each patient.

## 4 Results

A total of 24 eyes from 12 consecutive patients (8 females, 4 males) with superior sulcus hollowing were treated with i‐PRF injections. The mean age was 47.4 ± 8.5 years (range: 35–64), and the mean follow‐up duration was 12.8 ± 7.2 months (range: 6–24 months). Fitzpatrick skin types ranged from II to IV, with 5 patients classified as type II, 5 as type III, and 2 as type IV. All patients presented with moderate‐to‐deep hollowing, defined by pretarsal skin show > 7 mm.

**FIGURE 4:**
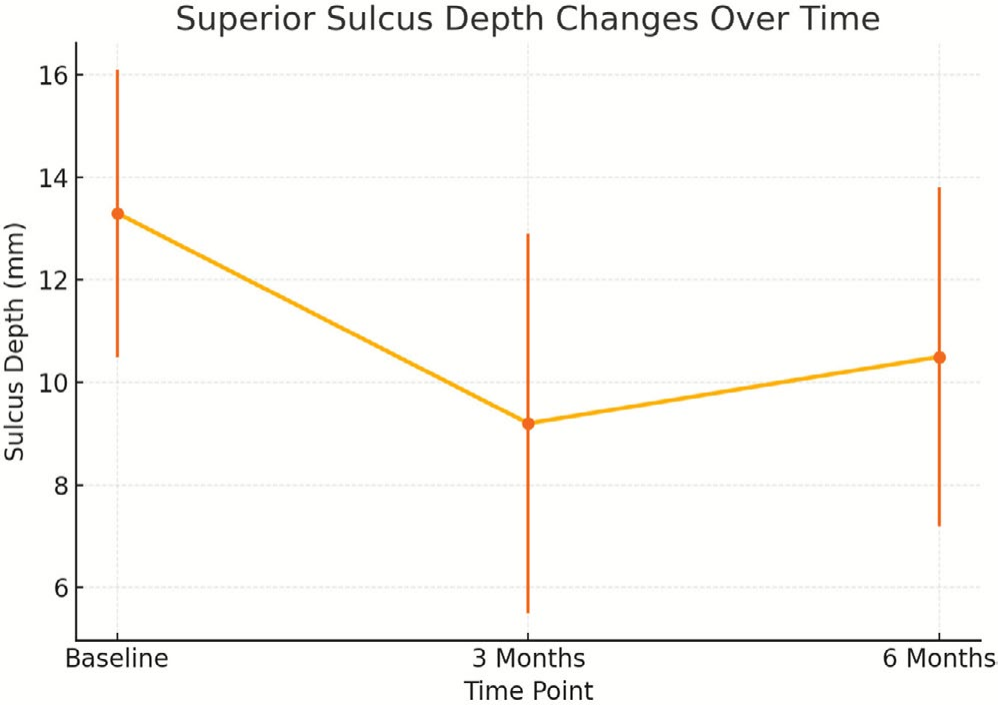
Superior sulcus depth changes over time following i‐PRF injections. Mean sulcus depth decreased significantly from baseline to 3 months, followed by a mild increase at 6 months. Error bars represent standard deviation (SD), illustrating interpatient variability.

Quantitative analysis showed a significant reduction in superior sulcus depth, with the greatest improvement at 3 months. Mean depth decreased from 13.3 ± 2.8 mm at baseline to 9.2 ± 3.7 mm at 3 months (*p* = 0.002 vs. baseline), followed by a mild increase to 10.5 ± 3.3 mm at 6 months (*p* = 0.01 vs. baseline). Figure 4 illustrates these changes over time in a line graph, enhancing the visual clarity of treatment outcomes.

**FIGURE 5:**
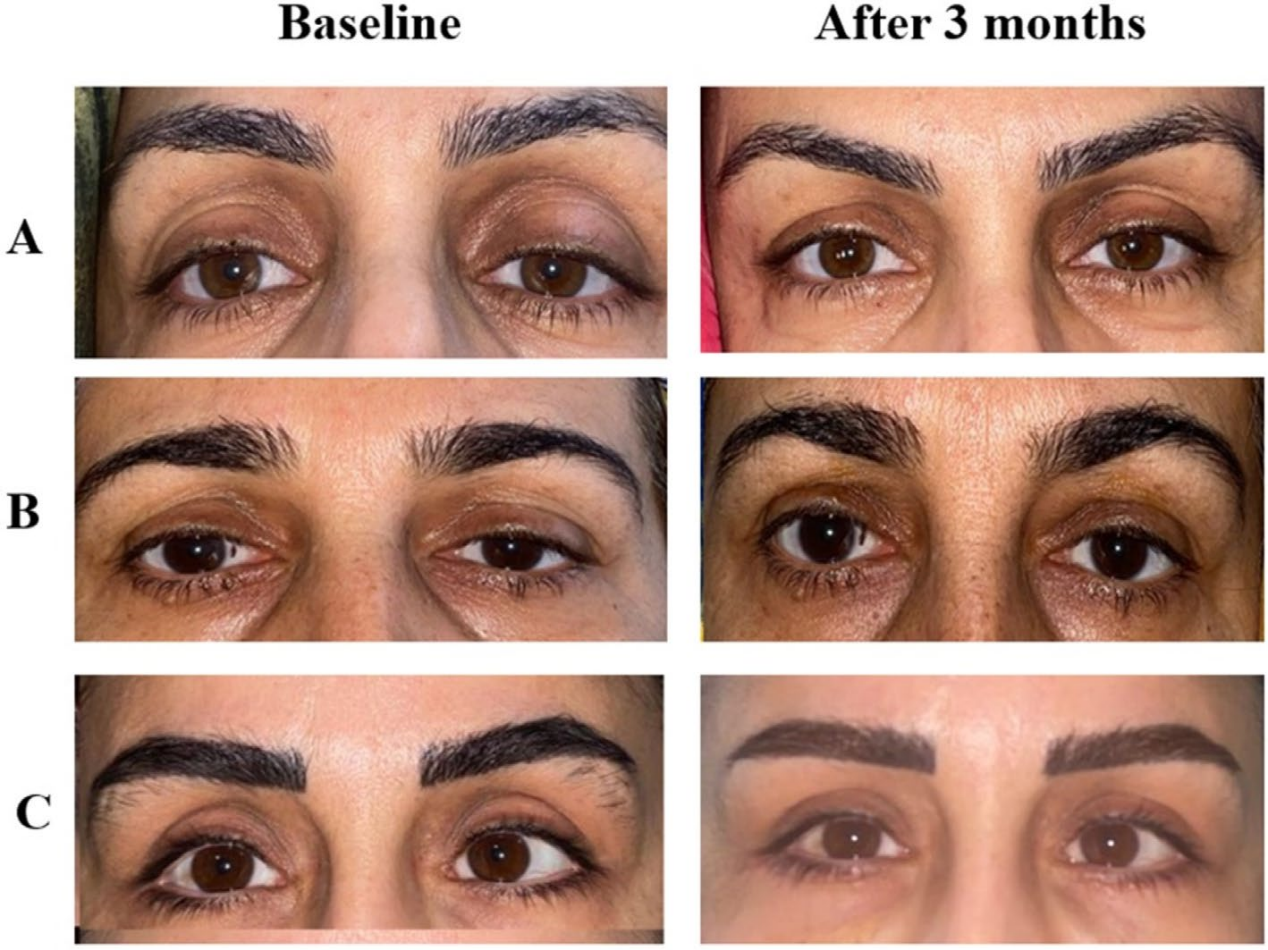
Pre‐ and posttreatment clinical photographs of patients undergoing i‐PRF injections for superior sulcus correction. Panels A–C represent four different patients, aligned horizontally with clear separation. Left column: Baseline appearance; Right column: 3‐month posttreatment results. Improvement in upper eyelid contour and sulcus volume is evident in all cases.

Patient satisfaction was reported in 18 of 24 eyes (75%) at 3 months and remained stable at 6 months. Transient edema and mild bruising occurred in 2 patients (16.7%), resolving spontaneously within 3 days, and no serious complications were observed. Representative pre‐ and posttreatment clinical photographs demonstrating improvement in upper eyelid contour and superior sulcus volume are shown in Figure 5.

## 5 Discussion

This study introduces a novel, biologically driven approach to superior sulcus rejuvenation by evaluating the clinical and esthetic efficacy of i‐PRF. Although traditional methods such as hyaluronic acid fillers, autologous fat grafting, and dermis‐fat implantation have been widely used, each carries potential risks including invasiveness, cost, and complications such as infection or even blindness [2, 3]. i‐PRF, a fully autologous and minimally invasive approach, may represent a promising alternative with a favorable safety profile.

PRF is a second‐generation platelet concentrate that retains platelets, leukocytes, and a fibrin scaffold without the use of anticoagulants [4, 5]. Compared to PRP, PRF has been shown to release higher levels of growth factors over a prolonged period, enhancing its regenerative capacity [13, 14]. Moreover, the injectable form of PRF (i‐PRF), developed by modifying centrifugation parameters, enables liquid delivery and in situ fibrin polymerization [6, 7, 8]. This structural fibrin matrix may enhance stem cell migration and tissue healing more effectively than PRP [7].

While PRF is commonly utilized in dentistry—for gingival recession, wound closure, and periodontal regeneration [15, 16, 17, 18]—its application in ophthalmology remains limited. Several recent studies have demonstrated its potential role in accelerating epithelial healing and reducing recurrence after pterygium surgery [9, 10, 19]. In esthetic medicine, intradermal i‐PRF injections have been associated with improved skin tone and texture, as shown by Hassan et al. in the nasolabial region and malar areas [20]. Similarly, Lee et al. [21] and Nacopoulos et al. [22] reported enhanced skin satisfaction and rejuvenation following i‐PRF use.

In the context of deep SS, age‐related or iatrogenic loss of orbital and submuscular fat leads to a hollowed upper eyelid appearance, often perceived as tired or aged [1, 3]. Previous reviews have summarized the spectrum of surgical and nonsurgical treatment approaches for deep superior sulcus and highlighted the importance of proper clinical evaluation and treatment selection [23]. Various classifications for SS have been proposed, such as the four‐pattern system by Morley et al. [24], and grading scales based on concavity depth [2, 25]. However, the lack of standardized numerical criteria and the influence of factors like blepharoptosis and skin redundancy limit their clinical utility. In our study, objective classification was not performed due to the limited sample size. Nevertheless, 75% of patients reported subjective satisfaction, and no complications were observed.

Among the 12 patients, three required additional injections 1 month after the final session. This highlights potential variability in response and the need for personalized treatment protocols. Compared to synthetic fillers, i‐PRF offers advantages such as ease of preparation, lower immunogenicity, and intrinsic regenerative properties. Its content of platelets, fibrin, and leukocytes contributes to anti‐inflammatory and reparative effects, making it a biologically favorable filler candidate.

This study has several limitations. The small sample size and retrospective design without a control group limit the generalizability of our findings. Patient satisfaction was assessed using a simple binary scale rather than a validated esthetic outcome measure, which may introduce subjectivity. Follow‐up duration varied from 6 to 24 months; while this reflects real‐world clinical practice, it introduces heterogeneity in outcome assessment.

Despite these limitations, the 6‐month follow‐up period exceeds the typical 3‐month assessment used in many esthetic studies and provides valuable insight into the short‐ to mid‐term efficacy of i‐PRF. Compared with established options such as hyaluronic acid fillers, fat grafting, or stromal vascular fraction gel [23, 26, 27], i‐PRF offers the advantages of autologous origin, regenerative potential, and minimal invasiveness. Future studies with larger patient groups should include standardized patient‐reported outcome assessments, objective volumetric imaging for quantifying tissue changes, and the use of ultrasound guidance to improve procedural safety. A key strength of the present study is its introduction of injectable platelet‐rich fibrin (i‐PRF) as a minimally invasive, autologous alternative for superior sulcus hollowing—an area where current literature is scarce. To our knowledge, this is the first clinical investigation specifically evaluating i‐PRF for the correction of a sunken upper eyelid. The study combined objective anatomical measurements with patient‐reported outcomes, providing a dual perspective on treatment efficacy. Moreover, the use of a standardized injection protocol and consistent follow‐up design enhances methodological clarity and reproducibility.

In conclusion, i‐PRF appears to be a promising, cost‐effective, and safe option for superior sulcus hollowing. Its autologous nature, ease of preparation, and regenerative potential offer distinct advantages over synthetic fillers and fat grafting. Despite the encouraging mid‐term results and high patient satisfaction rates, further large‐scale, prospective trials with standardized outcome measures are needed to confirm long‐term efficacy and to optimize treatment protocols. Given the anatomical complexity and potential risks of periocular injections, these procedures should be performed exclusively by experienced clinicians.

## Author Contributions

All authors contributed to the study conception and design. Material preparation, data collection, and analysis were performed by Nese Arslan, Alperen Bahar, and Ahmet Alp Bilgic. Şule Barman Kakil contributed to manuscript revision and interpretation of findings. The first draft was written by Nese Arslan, and all authors reviewed and approved the final version of the manuscript.

## Funding

The authors have nothing to report.

## Ethics Statement

The study was conducted in accordance with the Declaration of Helsinki and approved by the institutional ethics committee (Approval number: 2023‐292, dated 14/06/2023). Written informed consent was obtained from all participants.

## Conflicts of Interest

The authors declare no conflicts of interest.

## Data Availability

The data that support the findings of this study are available on request from the corresponding author. The data are not publicly available due to privacy or ethical restrictions.

## References

[1] A. Camirand , J. Doucet , and J. Harris , “Eyelid Aging: The Historical Evolution of Its Management,” Aesthetic Plastic Surgery 29, no. 2 (2005): 65–73. 15803355 10.1007/s00266-003-0066-1

[2] S. Park , B. Kim , and Y. Shin , “Correction of Superior Sulcus Deformity With Orbital Fat Anatomic Repositioning and Fat Graft Applied to Retro‐Orbicularis Oculi Fat for Asian Eyelids,” Aesthetic Plastic Surgery 35 (2011): 162–170. 20835821 10.1007/s00266-010-9574-y

[3] T.‐M. Lin , T.‐Y. Lin , C.‐K. Chou , C.‐S. Lai , and S.‐D. Lin , “Application of Microautologous Fat Transplantation in the Correction of Sunken Upper Eyelid,” Plastic and Reconstructive Surgery. Global Open 2, no. 11 (2014): e259. 25506542 10.1097/GOX.0000000000000141 PMC4255902

[4] J. Choukroun , F. Adda , C. Schoeffer , and A. Vervelle , “PRF: An Opportunity in Perio‐Implantology,” Implantodontie 42 (2000): 55–62.

[5] J. Choukroun and R. J. Miron , “Platelet Rich Fibrin: A Second‐Generation Platelet Concentrate,” in Platelet Rich Fibrin in Regenerative Dentistry: Biological Background and Clinical Indications: Biological Background and Clinical Indications (Wiley, 2017), 1–14.

[6] X. Wang , Y. Zhang , J. Choukroun , S. Ghanaati , and R. J. Miron , “Effects of an Injectable Platelet‐Rich Fibrin on Osteoblast Behavior and Bone Tissue Formation in Comparison to Platelet‐Rich Plasma,” Platelets 29, no. 1 (2018): 48–55. 28351189 10.1080/09537104.2017.1293807

[7] Y. Li , P. Song , J. He , et al., “Comparison Between Injectable Platelet‐Rich Fibrin and Platelet‐Rich Plasma in Ameliorating UVA‐Induced Photoaging in Human Dermal Fibroblasts via the Activation of TGF‐β/Smad Signaling Pathway,” Photochemistry and Photobiology 98, no. 6 (2022): 1395–1401. 35365859 10.1111/php.13628

[8] R. J. Miron , G. Zucchelli , M. A. Pikos , et al., “Use of Platelet‐Rich Fibrin in Regenerative Dentistry: A Systematic Review,” Clinical Oral Investigations 21 (2017): 1913–1927. 28551729 10.1007/s00784-017-2133-z

[9] C. Camacho and E. Rojas , “Platelet‐Rich Fibrin Membrane for Pterygium Surgery: Literature Review and Feasibility Assessment,” Cureus 13, no. 9 (2021): e17884. 34660083 10.7759/cureus.17884 PMC8503701

[10] A. Bahar and H. Sabur , “Effects of Injectable Platelet‐Rich Fibrin (i‐PRF) on Pterygium Surgery With Conjunctival Autograft,” International Ophthalmology 44, no. 1 (2024): 65. 38347311 10.1007/s10792-024-02920-5

[11] F. Romeo , “Upper Eyelid Filling With or Without Surgical Treatment,” Aesthetic Plastic Surgery 40 (2016): 223–235. 26893281 10.1007/s00266-016-0619-8

[12] C. F. Mourão , H. Valiense , E. R. Melo , N. B. Mourão , and M. D. Maia , “Obtention of Injectable Platelets Rich‐Fibrin (i‐PRF) and Its Polymerization With Bone Graft: Technical Note,” Revista do Colégio Brasileiro de Cirurgiões 42, no. 6 (2015): 421–423 English, Portuguese., 10.1590/0100-69912015006013. 26814997

[13] M. Fujioka‐Kobayashi , R. J. Miron , M. Hernandez , U. Kandalam , Y. Zhang , and J. Choukroun , “Optimized Platelet‐Rich Fibrin With the Low‐Speed Concept: Growth Factor Release, Biocompatibility, and Cellular Response,” Journal of Periodontology 88, no. 1 (2017): 112–121. 27587367 10.1902/jop.2016.160443

[14] R. Fijnheer , R. Pietersz , D. De Korte , et al., “Platelet Activation During Preparation of Platelet Concentrates: A Comparison of the Platelet‐Rich Plasma and the Buffy Coat Methods,” Transfusion 30, no. 7 (1990): 634–638. 2144922 10.1046/j.1537-2995.1990.30790385523.x

[15] S. Jankovic , Z. Aleksic , P. Klokkevold , et al., “Use of Platelet‐Rich Fibrin Membrane Following Treatment of Gingival Recession: A Randomized Clinical Trial,” International Journal of Periodontics & Restorative Dentistry 32, no. 2 (2012): 165. 22292152

[16] V. Jain , M. Triveni , A. T. Kumar , and D. S. Mehta , “Role of Platelet‐Rich‐Fibrin in Enhancing Palatal Wound Healing After Free Graft,” Contemporary Clinical Dentistry 3, no. Suppl 2 (2012): S240–S243. 23230372 10.4103/0976-237X.101105 PMC3514941

[17] H. Ajwani , S. Shetty , D. Gopalakrishnan , et al., “Comparative Evaluation of Platelet‐Rich Fibrin Biomaterial and Open Flap Debridement in the Treatment of Two and Three Wall Intrabony Defects,” Journal of International Oral Health: JIOH 7, no. 4 (2015): 32–37. 25954068 PMC4409793

[18] A. E. di Lauro , D. Abbate , B. Dell'Angelo , G. Iannaccone , F. Scotto , and G. Sammartino , “Soft Tissue Regeneration Using Leukocyte‐Platelet Rich Fibrin After Exeresis of Hyperplastic Gingival Lesions: Two Case Reports,” Journal of Medical Case Reports 9 (2015): 1–5. 26527036 10.1186/s13256-015-0714-5 PMC4630902

[19] N. Yang , Y. Xing , Q. Zhao , S. Zeng , J. Yang , and L. Du , “Application of Platelet‐Rich Fibrin Grafts Following Pterygium Excision,” International Journal of Clinical Practice 75, no. 10 (2021): e14560. 34155746 10.1111/ijcp.14560

[20] H. Hassan , D. J. Quinlan , and A. Ghanem , “Injectable Platelet‐Rich Fibrin for Facial Rejuvenation: A Prospective, Single‐Center Study,” Journal of Cosmetic Dermatology 19, no. 12 (2020): 3213–3221. 32852873 10.1111/jocd.13692

[21] Z. H. Lee , S. Sinno , G. Poudrier , et al., “Platelet Rich Plasma for Photodamaged Skin: A Pilot Study,” Journal of Cosmetic Dermatology 18, no. 1 (2019): 77–83. 29855132 10.1111/jocd.12676

[22] C. Nacopoulos , K. Gkouskou , D. Karypidis , et al., “Telomere Length and Genetic Variations Affecting Telomere Length as Biomarkers for Facial Regeneration With Platelet‐Rich Fibrin Based on the Low‐Speed Centrifugation Concept,” Journal of Cosmetic Dermatology 18, no. 1 (2019): 408–413. 29761887 10.1111/jocd.12666

[23] H. S. Moon , B. Ahn , J. H. Lee , D. K. Rah , and T. H. Park , “Rejuvenation of the Deep Superior Sulcus in the Eyelid,” Journal of Cosmetic Dermatology 15, no. 4 (2016): 458–468. 27095130 10.1111/jocd.12221

[24] A. M. Morley , M. Taban , R. Malhotra , and R. A. Goldberg , “Use of Hyaluronic Acid Gel for Upper Eyelid Filling and Contouring,” Ophthalmic Plastic and Reconstructive Surgery 25, no. 6 (2009): 440–444. 19935245 10.1097/IOP.0b013e3181b80eb8

[25] L. Liang , H. Sheha , Y. Fu , J. Liu , and S. C. Tseng , “Ocular Surface Morbidity in Eyes With Senile Sunken Upper Eyelids,” Ophthalmology 118, no. 12 (2011): 2487–2492. 21872934 10.1016/j.ophtha.2011.05.035

[26] Y. Sung , R. A. Goldberg , and H. Lew , “Periorbital Injection of Hyaluronic Acid Gel in Patients With Deep Superior Sulcus,” Journal of Craniofacial Surgery 31, no. 1 (2020): 271–273, 10.1097/SCS.0000000000006060. 31794448

[27] F. Ding , Y. Shen , L. Lu , et al., “Correction of Mild‐To‐Moderate Sunken Upper Eyelids of Asians With Stromal Vascular Fraction Gel,” Ophthalmology and Therapy 12, no. 1 (2023): 535–548, 10.1007/s40123-022-00615-7. 36510031 PMC9834495

